# Persevering professionals: dilemmas of relationships and self-determination in work with people with intellectual disability – a multi-method study based on interpersonal process recall

**DOI:** 10.1177/17446295231154126

**Published:** 2023-02-01

**Authors:** Mari Husabø, Magne Mæhle, Målfrid Råheim, Aud Marie Øien

**Affiliations:** Department of Global Public Health and Primary Care, University of Bergen, Norway; Department of Welfare and Participation, Western Norway University of Applied Sciences, Sogndal, Norway; Department of Welfare and Participation, RinggoldID:% 7376 Western Norway University of Applied Sciences, Sogndal, Norway; Department of Global Public Health and Primary Care, 60518 University of Bergen, Norway; Department of Welfare and Participation, RinggoldID:% 7376 Western Norway University of Applied Sciences, Sogndal, Norway

**Keywords:** interpersonal process recall, people with intellectual disability, professional practice, relational work, self-determination

## Abstract

The article focuses on social educators’ reflections on their own professional practice in encounters with people with intellectual disability receiving services. Drawing on Interpersonal Process Recall, a video-assisted method, together with a focus group interview, the study explores the experiences from in-situ encounters of five social educators employed in a Norwegian municipality. The key findings are that they view relationship-building as integral to their work, they grant primacy to the ideal of autonomy and they strive towards realizing this in their daily work. The study however displays how these emphases might lead to dilemmas, especially between the wish to support the service users’ self-determination and the urge to protect them from harm. Of special note was how the service users’ increasing use of social media was perceived as a particular challenge for social educators, who were left with an experience of being unable to protect.

## Introduction

Professional practice with people with intellectual disability receiving services has undergone major changes during the last thirty years. In line with the international human rights treaty The Convention on the Rights of People with Disabilities (CRPD) (United Nations, n.d.), Norwegian laws and conventions, as in most Euro-American societies, recognize the equal rights of all peoples with disabilities to live independently, have full participation and inclusion, equal opportunities, and accessibility in society. In Norwegian policy reforms, a rapid deinstitutionalization process in the 1990s (Hutchinson & Sandvin, 2019) was followed by increased attention to the importance of values centering on self-determination. The expression of self-determination is influenced by the interaction between personal characteristics and environmental conditions. Support and accommodation enabling the exercise of self-determination is especially important for adults with intellectual disabilities (Wehmeyer & Bolding, 2001; Wehmeyer & Garner, 2003). This calls for a relational understanding of self-determination conceptualized through *relational autonomy* (Mackenzie & Stoljar, 2000). This concept refers to a range of perspectives on autonomy in feminist theory highlighting the importance of interdependence and relationships, rather than independence, as preconditions for control and agency. The understanding of autonomy as emerging through the support and enablement of others (Davy, 2019) and the acknowledgement of the connection between the self and the context allows for a wider understanding of autonomy among oppressed individuals, useful in disability studies (e.g. Björnsdóttir et al., 2015; Dowling et al., 2019). The term fits well with the relational approach to disability, which takes precedence in the Nordic countries, where disability is understood as formed in the relation between a person and the human-caused environment (Tøssebro, 2009, 2013). Hence, disability is a relationship, situational and contextual, and autonomy of the self is constituted in and through relationships (Davy, 2019).

While previous studies on autonomy and self-determination for people with intellectual disability largely have focused on the perspective of parents and professionals, recent studies (e.g. Björnsdóttir et al., 2015; Chalachanová et al., 2021; Cudré-Mauroux et al., 2020; Dodevska & Vassos, 2013; Hutchinson & Sandvin, 2019; Kittelsaa, 2014; Nonnemacher & Bambara, 2011; Reisæter, 2021; Vaucher et al., 2019; Witsø & Hauger, 2020) have increasingly brought out voices of the people with intellectual disability. These studies confirm that good relationships with support staff can promote self-determination. To know, trust and like the support staff creates a context for all supportive actions to follow. The quality of relationships is crucial (Chalachanová et al., 2021; Cudré-Mauroux et al., 2020; Nonnemacher & Bambara, 2011), and professionals must be sensitive to the individual’s needs and wishes to create an atmosphere which encourages the communication of preferences and choices (Björnsdóttir et al., 2015). This requires both professional skills and knowledge as well as interpersonal skills (Dodevska & Vassos, 2013; Pallisera et al., 2018). In addition to providing new insights into the understandings and perceptions of people with intellectual disability, this literature contributes to development of professional practice. To date, however, research has paid little attention to *professionals’* perceptions of the role of relationships in their work with people with intellectual disability (cf. Hastings, 2010).

A distinguishing feature of professional practice with vulnerable service users is the tension between protecting them from harm while also supporting them to lead more independent lives (Saario et al., 2018). This dilemma is present in all services working with people with intellectual disability, and various studies explore how support staff manage this tension (see for example Hawkins et al., 2011; Mjøen & Kittelsaa, 2018; Wilson et al., 2008). Similar complexities are explored in the literature of intellectual disabilities within the anthropology of care, where studies have probed questions of dependency, autonomy, and moral dilemmas (such as McKearney, 2021; Pols et al., 2017). These perspectives, which also have ties to feminist theories, supplement understandings of the professional moral dilemmas, by questioning positive approaches to care, and portraying care as a terrain of ambivalence (McKearney, 2020; Thelen, 2021).

There are many examples of these dilemmas, such as related to alcohol use (Pols et al., 2017), medical issues (Wilson et al., 2008), and overeating (Hawkins et al., 2011). Another issue, of increasing saliency, is the use of Internet and social media. Taken together, studies find that people with intellectual disability are partly excluded from Internet use, but that some, despite barriers such as safeguarding concerns and language impairments, have positive experiences using social media (Caton & Chapman, 2016; Chadwick et al., 2013), and that social media skills can deepen and extend social networks (Raghavendra et al., 2018). The possible benefits of Internet use have received much less attention than the risks (Chadwick et al., 2013; Glencross et al., 2021), and despite limited knowledge, support staff view people with intellectual disability as a vulnerable group at risk from online dangers, while they themselves lack formal training to promote inclusion and cross the digital disability divide (Chiner et al., 2017, 2021; de Groot et al., 2022). Overall, these studies highlight the need for more research and professional training to support Internet use for people with intellectual disability. Moreover, the professionals’ challenge when navigating between supporting autonomy and protecting their service users online is so far unexplored.

## Aim of study

The study set out to explore qualified social educators’ experiences from in-situ practices. The primary aim was to explore social educators’ reflections on their own practice. However, because of their own emphasis on developing good relationships and supporting the service users' self-determination, this paper more specifically turns its attention to how the social educators face dilemmas between promoting independence and protecting their service users from potential harm.

## Design, material, and methods

### Research design

The study has a multi-method approach, taking Interpersonal Process Recall (IPR) as its starting point, followed by a focus group interview with the five participating social educators.

As a research method, IPR is a semi-structured individual interview based on video-assisted recall, focusing on the participant’s experiences as they occurred during a recorded session (Elliott & Shapiro, 1988; Larsen et al., 2008). We chose IPR in this study as it enables exploration of in-session interactions and makes conscious, unconscious, unspoken experiences from the interaction (cf. Larsen et al., 2008; Macaskie et al., 2015). The method provides possibilities for first-hand clarification from participants and allows mutual explorations and reflections between participants and researcher.

Adding to the strength of video-assisted recall, a focus group was chosen to continue the reflections from the IPR-sessions. The focus group allowed a joint conversation about the experiences both from the IPR-sessions and general practice. Whilst we applied this multi-method approach in a recent study of social workers within the Norwegian Labour and Welfare Administration (Husabø et al., 2022), and some studies use recordings to analyse interactions between vulnerable service users and support staff (Dowling et al., 2019; Saario et al., 2018), we are not aware of previous research that explore professional practice with people with intellectual disability through similar multi-methods.

### Sample

Health and care services for people with intellectual disability in Norway are mostly provided by municipal services which is the context for the present study. The participating professionals are all social educators, the official translation of the Norwegian title *vernepleiar*, also translated as learning disability nurse. As the professional practice is at the foreground, the people receiving services will throughout this paper be referred to primarily through the general term “service users”, in some cases people with intellectual disability or residents. When referring to staff in general, care workers or support staff are used.

The selection of participants was based on a combination of strategic and convenience sampling. Participants were recruited after initial contact with the municipal research unit and the responsible adviser in the agency for services for people with intellectual disability in a large Norwegian municipality. The adviser led the recruitment process and recruited social educators that worked in different parts of the agency’s services. The inclusion criteria were a bachelor’s degree in social education and at least five subsequent years of experience from professional practice. Three female and two male social educators aged 35 to 45 participated (*Maria, Ted, Eva, David, Sarah*). Participants’ experience with service users ranged from eight to sixteen years and included work with disability, geriatric care, psychiatry, and substance use.

The social educators recruited the five service users (*Maja, Tim, Eric, Dennis, Silje*), with inclusion criteria mild or moderate intellectual disability and the capacity to consent. Two were in their early twenties, two in their early thirties, the fifth was in her sixties. All had varied living conditions and different additional diagnoses, such as problems with addiction, psychiatric and somatic challenges. The recordings took place in different settings: two were recorded in residential facilities (“supported housings”), one in a day care centre during interaction in an arts and crafts activity, and two during weekly home-visits to service users who lived independently.

The Norwegian Centre for Research Data (NSD) approved the research project. Personal identifiable information is anonymised, the participants provided written consent, and names used in the paper are pseudonyms. The social educators were instructed to recruit service users whom they trusted could handle the video-recording of their meeting, and to whom they could explain what it meant to consent. Information letters were also repeated verbally. The service users were informed that their participation was voluntary and would not affect their services, and that they could withdraw at any time.

### Data development

The data material consists of audio-recordings and transcripts from the five IPR-interviews and audio-recordings, transcriptions, and field notes from the focus group interview. True to the IPR-method, the video-recordings were support material and not to be analysed separately.

The five individual IPR-interviews were each based on one video-recorded encounter between the social educator and their recruited service user. The first author conducted the interviews, and as a preparation for each interview roughly transcribed the recording. At the beginning of the individual interview, the social educator was introduced to a brief interview guide with a few fixed themes, such as working conditions, aims and expectations. Following the IPR-method, the interviews were mostly related to the video-recordings and conducted as close in time as possible, all within 48 hours. Creating a safe and trusting interviewing environment for the participants was important (cf. Larsen et al., 2008). The recording was played back for the social educators, and both the interviewee and researcher had the possibility to pause the recording and comment on specific sequences they found interesting, significant, or surprising, or where they wished to add something (cf. Macaskie et al., 2015). Addressing events as they occurred in the video-recorded session, the social educators’ experiences of what happened and their motivation for doing what they did (focus, questions, and comments), were explored. This in turn generated further reflections on their professional practice.

The social educators, the first and the fourth author participated in the subsequent focus group. Prior to the focus group, transcriptions were made of the five individual IPR-interviews, which allowed the researchers to address some preliminary themes. Due to the confidentiality, we could not discuss the video-recordings or the service users’ personal details. Each participant therefore retold what they experienced as most interesting and challenging from the IPR-session, while the researchers’ presented general patterns and common experiences. The dialogue in the focus group thus emerged both as continuations of reflections from the IPR-sessions and joint reflections on professional practice.

### Analysis

Data were analysed using an inductive, thematic analytic approach following six phases (cf. Braun & Clarke, 2006). The analysis concentrated on the social educators’ recall and reflections upon occurrences from the recorded encounters and the reflective dialogues that followed in the focus group.

During the first phase, the first author transcribed the six interviews, followed by readings and re-readings by the first and fourth author to familiarize with the data and form an overall impression. In the second phase, the first author conducted a broad initial coding which the first and fourth author subsequently discussed in the third phase, resulting in potential themes relevant for understanding the social educators’ experiences: *being professional, dilemmas of autonomy, being responsible, tools and possibilities in the conversations, importance of knowing, outside forces, friendship, challenging social media*. In the fourth phase, the potential themes were checked both in relation to the coded extracts and the entire dataset to ensure validity, with emphasis on the questions of autonomy and relational work. In the fifth phase of the analysis, the first author selected extract examples from the material to illustrate themes and analytical points, before all authors participated in defining, refining, and naming the themes. This prepared the final write-up of the report in the sixth phase, relating the analysis to the research question and literature.

The analysis yielded two core themes: “Dilemmas of relationships” and “Dilemmas of self- determination”, including subthemes and extract examples.

### Methodological considerations

Research involving people with intellectual disability in the empirical material raises special ethical concerns related to information, sampling, and consent, and requires heightened awareness (Cudré-Mauroux et al., 2020). The potential vulnerability and reduced cognitive abilities can also amplify the uneven power relations between researchers and participants (van der Weele & Bredewold, 2021). However, since the participating service users did not directly interact with the researchers, these issues were less pertinent. The social educators prepared their service users for the video-recordings and offered them to talk about the participation afterwards. The service users expressed a positive experience of participating in the project.

The outbreak of covid-19 and extensive restrictions in the municipal services excluded the researchers from doing data development for nine months. This prolonged process led to participant dropout and further delays, and some of the participants found the gap in time between the IPR-sessions and focus group long. While this made it difficult to remember the details of the IPR-session and thus access the recalls, it allowed a process of raised awareness on the cooperation with the particular service user.

Following the thematic approach, the data were analysed as a whole. As the IPR-sessions and hence actual practical experience lay the foundation for the focus group, the findings from the two sources are not systematically differentiated. Joint analysis with co-authors, in our case with three senior researchers holding vast differentiated clinical experience is conducive to reliable findings. Furthermore, researchers with educational background and experience from working with people with intellectual disability considered the findings to be recognizable supporting pragmatic validity.

## Findings

The analysis identified how the social educators especially valued two dimensions of their work with their service users, namely the foundational role of relationships and the importance of supporting self-determination. These values were however also related to a range of dilemmas that are explored in the themes and subthemes that follows.

### Dilemmas of relationships

The social educators’ emphasis on relationship stands out as a key finding of this study. Getting to know the users, learning which approach worked best for them and continually crafting the relationships were considered to be their core business as well as the starting point for working with more delicate and difficult issues and challenges. This was stated several times both in the IPR-sessions and in the focus group discussion, as by Maria, who claimed that 90% of their work consisted of working with what she termed “the good relation” and keeping up the service users’ spirits.

According to the social educators, “getting to know” was time-consuming work, built purposely piece by piece. While the frequency of visits and time spent together differed between those working in supported housing and those in home-care services, all five concurred that the most important source of knowledge was the individual service user. They also emphasized interpersonal chemistry and strived to find common areas of interest - spending time together, talking, and performing activities such as basic household chores, walking or game play, was experienced as vital in “getting to know”, and often served as entry points into more sensitive issues. The intimate knowledge of the user entailed seeing “the whole person”, including diagnoses, compound challenges and awareness of physical signals, conditioned by the day-to-day situation. This was especially important in work with users suffering from severe medical conditions, such as for Sarah, whose user Silje suffered from a disease that caused frequent and often sudden epileptic seizures. During the videorecording Silje had an epileptic seizure, and in the following interview Sarah recalled the physical signals ahead of the seizure, as well as possible triggers during the last 24 hours.

#### Relational dilemmas: Consequences of “not knowing “the service users well enough

The emphasis on “knowing” could however lead to challenges, as the feeling of “not knowing” the service users sufficiently seemed to lead to uncertainty regarding recourses and needs for assistance. This was illustrated in a peculiar way with Ted, who did not know his service user Tim very well. During the videorecording, Tim agreed to everything Ted said and seldom replied more than “yes” to any question. According to Ted, this was business as usual. Tim had just recently moved away from home, and his meetings with the home-care services were restricted to thirty minutes twice a week. Tim’s aloofness to visitors sustained their experience of “not knowing” him, and six months after the IPR-session, in the postponed focus group session, Ted related that Tim now mostly canceled one of the two weekly visits. Ted found it difficult to evaluate Tim’s functional level and to decide whether they should assist him in getting a workfare or engage in day activities. He was also unsure whether Tim appreciated their visits or if he just played along and wanted to be left alone. Ted revisited this deadlock; because of the limited knowledge they had of Tim, their possible approaches were limited, and further relationship-building got impaired. Additionally, other factors, such as lack of resources and understaffing, could also impede relational work.

#### Relational dilemma: Drawing the lines between friendship and professional relationships

Despite the emphasis on common interests and interpersonal chemistry, the social educators relentlessly underlined important differences between friendships and professional relations, as explained by David: “no matter how you twist and turn - when I walk out of here, I leave work behind. And once a month I get paid for being here. And at the end of the day, I must make decisions that a friend doesn´t have to, and I have to give messages that a friend wouldn´t give”. However, ambivalences appeared several times. “We can´t pretend in the relation but must care and be interested for real. If we don´t, they get it”, Maria argued. “We go through all feelings together, you know – a bit like we do with our own children”. The social educators experienced getting emotionally attached to service users, and, despite change of jobs and thus a breaking up of relations, kept them in their hearts.

The social educators were also conscious of the numerous relationships that their service users were part of, such as Dennis, who according to David related to more than thirty staff in the supported housing, besides neighbours, staff, and colleagues at the supported employment. David and Sarah pointed to the ethical dilemma: “We are constantly facing choices when it comes to relations – we go all in to get to know a person, to build relations. And then, afterwards, we fade away. What happens to these people, having to meet all these carers throughout their lifetime? Imagine the number of relations – and grieving processes - through the years. No wonder if that make them hard to get close to and leaves them pondering: Who are really my friends?”. The social educators called for more knowledge on how to secure continuous care for service users that had to handle countless relationships and break-ups during their lifetime.

#### Relational dilemma: The service users right to choose - or single out the relationships

While the service users in some cases had to accept certain invasions in their personal sphere others had greater possibilities to navigate, select, and refuse the services they were offered. This underscored the importance for the social educators of developing good relations, not only as the fundaments of the rest of their work, but also as the very door opener towards the service users. Ted illustrated this with his hard work “getting to know” Tim: “the present problem is to be allowed to visit him twice a week. I try to strike a friendly chord. If he doesn’t feel that the relationship is good…., then we won’t be allowed to enter at all…”. Sarah continued: “…and if they slam that door, it stays closed and it can take ages to re-enter”. Keeping the relation going was therefore imperative, as possible intermissions in the relationship could mean having to start all over again. This indicates a perpetual challenge for the social educators: The service users’ right to determine what services to receive and what relationships to enter – or leave.

### Dilemmas of self-determination

Another key finding is how the social educators emphasized the service users’ right to govern their own life. The ideal of autonomy was considered fundamental to practice and profession, and the strive towards realizing this ideal seemed to be embedded in their daily work. This was thoroughly stressed by Sarah, when challenged on why she placed such importance on Silje’s participation in minute detail: “It is her life, her everyday! Of course, you have a wish that all humans could be autonomous, independent and govern their own life! We are not supposed to dictate how they should choose [to] live their lives. That is the human rights act, isn’t it!”

Advocating for the service users’ needs and furthering their desires were understood as a professional imperative, as described by Maria: “[as a social educator] my main mission remains: helping the service users to be heard”. The social educators were emphatic about listening to the service users and understanding their desires. Further, they regarded it as their task to lay the groundwork for the service users to make their own decisions, take care of their interests towards other municipal services, next of kin and the larger society, and make sure that their voices were heard in cases of conflicting interests. Additionally, they emphasized their advisory role and the importance of not acting imperiously or denying the service users autonomy. Rather, service users should know that they were allowed to reject. During the IPR-sessions countless examples appeared, ranging from choice of yoghurt -flavors and day activity to whether to apply for an extra weekly shower from the home nursing care. The risk of marginalization of their service users, due to linguistic or cognitive challenges, was a constant concern for the social educators, who also feared that the social distancing, isolation and lock-down brought on by the covid-19 pandemic could lead to increased marginalization of people with intellectual disability and affect the development of self-determination negatively.^1^

#### Dilemmas of self-determination: supporting autonomy while protecting from harm

However, despite the indisputable consensus regarding and sensitivity towards the importance of self-determination, several dilemmas emerged. The most basic and recurring was the dilemma between respecting the service users’ right to govern their own life and following the urge to act according to what the social educators termed as “the best interest of the user”. As the service users had compound physical, psychological and social challenges, the social educators continually faced situations that demanded careful considerations and attempts to lead the service users away from what they considered to be “worse decisions” towards better ones. All five identified situations in the video-recorded material where such dilemma occurred, ranging from minor questions – such as whether to insist that the service users dressed according to the weather - to more serious matters, as in Silje’s case, where her severe medical situation demanded Sarah’s physical presence. Dilemmas of self-determination were also present in the relational dilemmas, as when David reflected on Dennis’ numerous and partly involuntary relationships and in Ted’s puzzling over whether Tim wanted to accept visits from the municipal services at all.

One example came from Maria and her service user Maja, an elderly woman with an acquired brain injury from the early childhood years. Maja had a strong will, and an even stronger desire to take care of herself, despite physical disablements and limited mobility. She had however endangered herself several times, and during the video-recorded session, Maria tried to address the possible hindrances in Maja’s current housing situation. Maja abruptly changed the theme, laughed off and refused to answer. When Maria carefully reminded her about a GPS-alarm acquired in order to secure Maja access to 24 hours help, Maja swiftly faced the first author, who was present in the room, and jokingly exclaimed: “I’ll throw that dingus through the window!”. In the succeeding interview Maria addressed this as the most difficult and urgent dilemma regarding this service user. While both the home-care services and next of kin worried for her safety and wanted Maja to move to a supported housing, the most important matter for Maja was to continue taking care of herself, and she resisted anything she experienced as an attempt to restrain her. The present solution, Maria stated, was therefore to accept Maja’s wish to stay at home and rather furnish the apartment with remedies.

Another example appeared between Eva and her service user Eric, a man in his thirties with Down’s syndrome. In the video-recorded session Eva and Eric talked about the upcoming New Year’s Eve and New Year´s resolutions. Eric declared that he would become a “yes-boy” and tell the truth. And then, he added, “it is important to say sorry and I apologize if I have done anything I shouldn’t have”. Eva responded that she knew the staff sometimes could appear nagging, but that in the end they only wanted him to have the best life possible. In the subsequent IPR-interview Eva explained: Eric had a history of alcohol use and both staff and next of kin meant that the drinking was excessive and worsening his recurrent depressive disorder. As Eric had the capacity to consent, and, hence, the right to buy and consume alcohol, the staff had proposed a deal: Every Friday evening they should accompany Eric to limit the amount of beer he bought. Eric agreed and they signed the deal. Still, every Friday on his way home from work, Eric would buy beer that exceeded the written agreement. If confronted by the staff, he apologized, promised to change for the better, before repeating the deed once again the following week. Therefore, Eva sighed, she had little faith in his New Year’s resolution, but nevertheless felt that it was important to remind him that they had his best interest in mind.

#### Dilemmas of self-determination: social media and the potential online dangers

Another pressing dilemma resulted from the service users increasing access to, and use of, social media. All five social educators worried about the growing use of smartphones and other gadgets. Many of the service users had inadequate linguistic comprehension and encountered a lot of misunderstandings in their digital communication. The social educators retold of endless hours spent on conciliation between service users, due to misunderstandings often born out of spelling mistakes. Similarly, the service users’ difficulties related to friendship intensified in social media, both by the introduction of new relationships such as cyber acquaintances and by altering and adding new layers in existing relationships. Social media also added to the dilemmas of drawing the lines between professional relationships and friendships for the social educators, as the service users texted them outside workhours and sent friend requests through social media platforms. The social educators experienced their service users as exhausted from social media, lacking the proper tools or qualifications to comprehend and navigate the digital landscape. “It has become an extra factor,” Maria stated, “and they never get a break – the information keeps coming, and often they don’t have the ability to manage it”. Additionally, the social educators felt that they lacked professional competences and measures to advise their service users.

The social educators also found that existing regulations left little room for intervening in the service users’ use of social media. As an initiative to reduce stress and lack of sleep due to disturbance from social media, the staff at Eva’s workplace had, in agreement with the residents and next of kin, arranged for the residents to hand in their gadgets by bedtime and have them returned the next morning. However, despite immediately showing positive effects the initiative was stopped after an inspection from the authorities, as it was determined to breach legal safeguards. Coupled with the service users limited capacity to understand their repeatedly destructive patterns, the social educators felt trapped in the dilemma between wanting to protect their service users from the negative effects of social media and the ideal of supporting and enabling their self-determination.

The social educators also experienced a darker side of the service users’ use of social media. Due to the limited ability to understand both communication and nuances, service users often became victims of economic exploitation. Some had worsened mental illnesses or got negatively affected by extreme or radical opinions and groups, while others got criminalized, for example by stalking celebrities or distributing injurious pictures. In one of the supported housings, medical students from a nearby university had delivered lectures for the residents on topics related to sexuality and social media, but the staff experienced that the residents soon forgot the lessons and returned to their old pattern. A severe example of the powerlessness was offered by Maria, as she told the story of a young female service user whose use of dating apps repeatedly had resulted in severe violent sexual assaults. “Again, the self-determination … I am an ardent follower of the service users right to make, and learn from, their own mistakes. But still, there must be some limits. But I feel paralysed – we send notes of concern to the GP, or to the governor, but nothing happens, and we have to witness these terrible happenings, again and again”.

## Discussion

In this study, we set out to explore educated social educators’ reflections on their own practice in work with people with intellectual disability receiving services. We found that they insisted on the importance of relationships and granted primacy to the ideal of autonomy in their work with the service users. This echoes earlier studies (c.f. Björnsdóttir et al., 2015; Cudré-Mauroux et al., 2020; Dowling et al., 2019; Nonnemacher & Bambara, 2011) that emphasise the interconnectedness between these dimensions in work with people with intellectual disability. The social educators’ perspectives on how to support and enable the service users’ self-determination found in the present study seem to be consistent with the idea of relational autonomy (Davy, 2019). The findings also demonstrate how the social educators perceived relationships as constitutive for their professional practice and how they underscored the importance of getting to know the users and understand their needs and desires. This accords with the perspectives of people with intellectual disability receiving services identified in earlier studies: emotional support was most highly valued, together with interpersonal skills such as listening ability, patience and respect (Pallisera et al., 2018). In relationships perceived as positive, the service users seemed open to staff support, requested needed assistance and shared sensitive information (Cudré-Mauroux et al., 2020; Nonnemacher & Bambara, 2011). We find this congruence between the perspectives of the social educators in our study and those of the service users in previous studies promising for the social educators’ ambition of advocating for the service users’ needs, raising their voices, and supporting the ideal of autonomy.

While our findings contrast those of some earlier studies, which found support staff placing less emphasis on interpersonal skills and emotional support than the users (Dodevska & Vassos, 2013; Pallisera et al., 2018), the social educators’ underlining of relationships mirrors the findings of Pols et al (2017). They found that caregivers described how getting to know people, learning which approach worked best for a given client and constantly crafting relationships were their core business, and a necessary starting point for addressing problems and negotiating limits. Our findings additionally suggest that the social educators risk getting checkmated by their preoccupation with relational work, as in Ted’s case: When failing to obtain the desired relationship with Tim, Ted had the experience of failing in his overall care towards him. As such, the strong emphasis on relationships might cause unexpected dilemmas for the social educators. Interestingly, this furthermore serves as an illustration of a relational understanding of autonomy: just like the autonomy of people with intellectual disability depend upon the relationship of their carers (Björnsdóttir et al., 2015; Witsø & Hauger, 2020), so do the service users influence their carers (cf. Davy, 2019; Pols et al., 2017). The social educators’ emphasis of relationships can further be understood in light of the precedence of relational approaches in the Nordic countries (Tøssebro, 2009) and how the social educators experienced relations as integral to their professional education and identity (Folkman et al., 2019).

The most striking finding in this study, is the presence of a range of professional dilemmas. In line with findings in previous research (e.g. Hawkins et al., 2011; McKearney, 2021; Mjøen & Kittelsaa, 2018; Pols et al., 2017; Wilson et al., 2008), the social educators frequently experienced incidents where they considered the service users’ choices as negative for their own well-being or challenging for the social educators’ attempts to fulfil their professional responsibilities. The dilemma between support of autonomy and protection from harm was ever-present, and the examples of Maja and Eric display how the social educators tried to navigate this dilemma, attempting to persuade and lead the service users away from “worse decisions” towards better ones. The case of Maja also display how they strived towards realizing the ideal of autonomy in practice. Despite worrying for Maja’s safety in her current housing, Maria accepted her right to decide for herself, and tried to secure Maja’s safety by different measures. From an outsider-view, the acceptance of Maja’s wish to stay at home might be perceived as laissez-faire and a failure in the protection of a vulnerable individual (cf. Mjøen & Kittelsaa, 2018). However, understood through a relational approach, Maria adapts the intervention to Maja’s expressed desires, and enables her self-determination by equipping her current home as well as possible. Maria’s ambition to gradually change Maja’s conviction can moreover be understood through the relational understanding of autonomy: we all interfere with one another’s autonomy, influence one another and try to persuade others to see things our way (Davy, 2019; Pols et al., 2017). Maria’s actions also suggest a perseverance in work: she does not resign or suggest a definitive solution, but rather reflects on the continuous dilemma while simultaneously acting on the current situation.

Eric’s case however opens for an alternative understanding. While Pols et al (2017) emphasize the persuasion and interdependence between clients and caregivers also in cases of tension related to overuse of alcohol or drugs, McKearney (2021) argues that persuasive care is part of an ideology that frames individuals with disabilities as incapable and lacking agency. The “misfitting” of people with intellectual disability is not because they are dependent and vulnerable, but rather the opposite: they are too independent minded for the form of dependence they are repeatedly persuaded into (McKearney, 2021). Eric’s continual breach of agreement can be viewed as resistance to the offered relations of care. His independence and “unruly behavior” create a “misfit” in the relationship with Eva and her colleagues. Similarly, one can view Tim’s reluctance to the services offered by Ted as a resistance: Tim does not have the receptiveness for the caring attention and persuasion that is assumed by the social educators.

Turning to the ambivalence in the border between professional relationships and friendships, Maria’s comparison of her relationship with the service users to that of her own children serve as clear telling of emotionally charged relationships. This finding is consistent with earlier studies (Hastings, 2010; Wilson et al., 2008) that find support staff experiencing intense emotions and perceiving the relationships as affective and meaningful. However, such emotional perceptions might evoke strong feelings concerning the limits of their professional role (Wilson et al., 2008), as in our findings, when the lack of resources worked as impediments to the relational work the social educators pursued. Moreover, our findings indicate other ethical challenges related to relationships, as in David and Sarah’s reflections on the possible consequences of the continuous flow of carers entering and leaving their service users’ life. Although a few recent studies (Reisæter, 2021; Witsø & Hauger, 2020) briefly touch upon the strong feelings and deep sorrow changes in staff group might evoke, the negative outcomes of manifold relationships and breakups for people with intellectual disability have received little attention in research. The social educators’ call for more knowledge on how to understand and work with such challenges therefore seems pertinent both to practice and future research.

The tension between protecting service users from harm while also supporting them to lead more independent lives lie at the heart of work with vulnerable people (Hawkins et al., 2011; Saario et al., 2018). Wilson et al (2008) show how professionals felt a pressure to find definitive solutions to ethical dilemmas in work with people with intellectual disability, even though such solutions do not exist. The findings in our study suggest that continuous ethical reflections are part and parcel of professional work with people with intellectual disability. We agree with the idea of supporting professionals in acknowledging this existential reality of such dilemmas (cf. Wilson et al., 2008), and to offer opportunities to reflect on how to further promote and support self-determination (cf. Vaucher et al., 2019).

Considering the vast experience of facing and navigating ethical dilemmas, such as those related to medical conditions and alcohol use, an unanticipated finding is how the challenges relating to service users’ increasing use of social media seemed to overwhelm the social educators. Their lack of knowledge and training to support their service users in their online lives support evidence from previous studies (Chadwick et al., 2013; Chiner et al., 2017, 2021; de Groot et al., 2022; Glencross et al., 2021). Additionally, with the existing regulations leaving few opportunities to intervene in the service users’ digital lives, the social educators experienced failing in navigating the tension between respecting the service users’ right to autonomy and protecting them from online risks. In this context, the incidence of sexual assault resulting from online contact serve as a grave illustration of how the social educators are rendered incapable to protect vulnerable service users, even though the danger is known. In contrast to the continuous dilemmas described earlier, where they displayed perseverance in facing them, the dilemmas originating from the users’ online lives seemed to be experienced as unbearable to the social educators. Further work is required to develop professional support of Internet use for people with intellectual disability. There is also a need for more research to understand the professional dilemmas that might appear from vulnerable service users’ interaction in social media, as well as what strategies the professionals might apply when facing them.

## Conclusion

Through a multi-method approach with a focus on recall and reflection, the study allowed for an in-depth exploration of the social educators’ perceptions of their own practice, challenges, and dilemmas. The study supports previous research that emphasizes the interconnectedness between relationship building and the support of autonomy in professional practice with people with intellectual disability. Our findings of how the social educators perceived relationship as imperative to their work add to the understanding of the interconnectedness and interdependency inherent in professional practice with vulnerable service users. We support the call for more in-depth studies of the diverse and ambivalent relational aspects of the professional practice.

The study’s explorative approach encouraged the participants to reflect deeply on their work. This may explain why they to a great extent focused on ethical challenges and dilemmas, which have received special attention in this paper. We hesitate to view these experienced dilemmas as ethical problems that need to be solved once and for all. Rather, the article argues in favour of understanding them as continuous dilemmas, and thus as ongoing ethical discussions vital to professional work in this field. Instead of fighting, resisting, or giving in to these dilemmas, the participating social educators navigate, act, and persevere. However, the service users’ access to, and use of new technologies and social media have actualized the dilemma between self-determination and care of people with intellectual disability, at worst rendering the social educators powerless while their service users’ risk being criminalized or subjected to abuse. Future research should be undertaken to explore both how to support vulnerable people’s Internet use and how to support the professionals that work with them.

## Limitations

This study is limited to exploring the professionals’ experiences; thus, the service users’ experiences are left unexplored. In addition to our call for further research on Internet use for vulnerable people, a natural progression of this study is therefore to explore and analyse service users’ experiences from similar encounters.

## Note

1. The extensive restrictions and closedowns that hit services and institutions during the pandemic did not happen without controversy. Local Norwegian authorities were criticized for sidelining a large group of inhabitants, infringing their autonomy and right of self-determination. For more on this discussion, see for example https://www.vg.no/nyheter/innenriks/i/86w9r2/besoeks-forbudet and https://fontene.no/debatt/kommuners-besoksstans-under-pandemien-viser-at-personer-med-utviklingshemming-forstas-som-ting-ikke-som-mennesker-6.47.732598.af5732da9d
